# Hypoxia enhances cholangiocarcinoma invasion through activation of hepatocyte growth factor receptor and the extracellular signal-regulated kinase signaling pathway

**DOI:** 10.3892/mmr.2015.3865

**Published:** 2015-05-27

**Authors:** THITINEE VANICHAPOL, KAWIN LEELAWAT, SURADEJ HONGENG

**Affiliations:** 1 Department of Molecular Medicine, Faculty of Science, Mahidol University, Bangkok 10400, Thailand; 2 Department of Surgery, Rajavithi Hospital, Mahidol University, Bangkok 10400, Thailand; 3 Department of Pediatrics, Ramathibodi Hospital, Mahidol University, Bangkok 10400, Thailand

**Keywords:** hypoxia, cholangiocarcinoma, invasion, metastasis, extracellular signal-regulated kinase 1/2, hepatocyte growth factor receptor

## Abstract

Hypoxia is associated with tumor progression and poor prognosis in several cancer types. The present study aimed to examine the contribution of hypoxia (1% O_2_) to cancer progression in a cholangiocarcinoma cell line, RMCCA-1. The molecular basis of the hypoxic response pathway was investigated. The results showed that hypoxia significantly accelerated cancer cell proliferation and enhanced cell invasion (P<0.05). By using receptor tyrosine kinase and intracellular signaling antibody array kits, an increased phosphorylation/activation of a number of signaling molecules, particularly hepatocyte growth factor receptor (Met) and extracellular signal-regulated kinase (ERK) 1/2, was identified. Inhibition of Met and ERK by small hairpin RNA and U0126, respectively, significantly inhibited hypoxia-induced the invasive potential of RMCCA-1 cells (P<0.05). However, according to immunohistochemical analysis, hypoxia-inducible factor-1α expression was not correlated with cancer staging or tumor differentiation in 44 samples of cholangicarcinoma cases. The findings of the present study emphasized the importance of Met/ERK pathway activation as a key molecular event that may be responsible for a more invasive phenotype in hypoxic tumors and suggest Met as a potential target for the treatment of cholangiocarcinoma.

## Introduction

Cholangiocarcinoma is an aggressive and lethal disease arising from the epithelial cells in the intra- and extrahepatic bile ducts. Although the annual incidence of cholangiocarcinoma is relatively low with 1–2 cases per 100,000 in western countries (1), it greatly varies among the population. The incidence is as high as 113 per 100,000 in Southeast Asia, with particularly high rates in Thailand. This is possibly due to liver fluke infection as an important risk factor (2). The cancer is highly resistant to chemotherapy with a high recurrence rate (3). To date, surgical resection has been the main treatment option. A five-year survival rate of only 25–30% has been reported among those who have had successful resection (4). Thus, an understanding of tumor biology underlying an aggressive phenotype of cholangiocarcinoma is crucial for the development of alternative treatments to improve clinical outcomes.

Inadequate supplies of oxygen as a result of rapid cell growth and aberrant vasculature formation are commonly found within most solid tumors (5). There is accumulating evidence suggesting that hypoxia contributes to tumor progression and metastasis by activating diverse signaling pathways (6,7). Tumor hypoxia has been associated with increased malignancy and poor prognosis (8). Elevated expression of hypoxia-inducible factor-1α (HIF-1α), a key transcriptional regulator in hypoxia, is correlated with a low survival rate in a wide variety of cancers (9). Numerous *in vitro* studies have shown that hypoxia can promote angiogenesis, resistance to chemotherapy and cell invasion (5–11).

Neoplastic malignancies are usually controlled by growth factors. Overexpression and overactivation of hepatocyte growth factor (HGF) receptor (Met) receptor tyrosine kinase (RTK) is strongly linked to oncogenesis and invasive growth (12,13). The receptor has a role in cell motility through activation of the mitogen-activated protein kinase (MAPK)/extracellular signal-regulated kinase (ERK) and PI3K/Akt pathways as its main downstream signaling axes upon hepatocyte growth factor (HGF) binding (14). Met induction by HGF was reported to have an important role in cholangiocarcinoma invasion ability (15). Penacchietti *et al* (11) showed that hypoxia can induce Met expression in a number of cancer cell types, including hepatocarcinoma, lung and ovarian carcinomas. Moreover, the MAPK/ERK pathway, containing downstream effectors of Met, regulates multiple cellular functions, including proliferation, cell motility and apoptosis (16), and hyperactivation of this pathway is a hallmark of cancer (17). The ERKs have a role in hypoxic conditions by regulating HIF-1 expression and function (18,19), which in turn regulates transcription of several genes involved in proliferation, glycolysis, angiogenesis and cell motility (5). HIF-1 is a heterodimeric basic helix-loop-helix transcription factor consisting of oxygen-dependent α- and constitutively expressed β-subunits. During hypoxia, the subunits dimerize to modulate gene expression.

Although a number of hypoxia-associated proteins involved in tumor progression have been well characterized in numerous types of cancers, the hypoxia-mediated pathways in cholangiocarcinoma remain mostly unknown. The present study was designed to examine effects of hypoxia on cholangiocarcinoma growth and its invasive potential, and to investigate the molecular basis of cancer invasion triggered by chronic hypoxia using a cholangiocarcinoma cell line.

## Materials and methods

### Cell culture

The human cholangiocarcinoma cell line RMCCA-1 (established at the Department of Surgery, Rajavithi Hospital, Bangkok, Thailand) (3), was cultured in HAM's F12 medium (Gibco-BRL, Life Technology, Carlsbad, CA, USA) supplemented with 10% fetal bovine serum (FBS; GE Healthcare Life Sciences, Logan, UT, USA) and 1% penicillin/streptomycin at 37°C in a 5% CO_2_ humidified atmosphere for normoxic conditions. Incubation under hypoxic conditions was performed under 1% O_2_ at 37°C in a hypoxic chamber (Stem Cell Technologies, Vancouver, BC, Canada).

### Immunofluorescence staining

Cells were plated and cultured on glass cover slips under hypoxic conditions for 48 h. Cells were then fixed with 4% paraformaldehyde (Sigma-Aldrich, St. Louis, MO, USA), permeabilized with 1% Triton X-100 (Sigma-Aldrich) and blocked with 1% bovine serum albumin (United States Biological, Salem, MA, USA). Rabbit monoclonal antibody against HIF-1α was used (ab51608; 1:100 dilution; Abcam, Cambridge, UK), followed by incubation with Alexa Fluor^®^ 488-labeled anti-rabbit secondary antibody (A-11034; 1:200 dilution; Thermo Fisher Scientific, Waltham, MA, USA).

### Cell proliferation assay

Cells were seeded in a 96-well culture plate at a density of 1,000 cells/well. Cell proliferation was assessed on days 1, 3 and 5 post-plating using a WST-1 Cell Proliferation Assay (Roche Diagnostics, Basel, Switzerland) following the manufacturer's instructions. Cells were incubated in a hypoxic chamber, which was re-purged every 24 h. The medium was replaced every other day. Absorbance was measured at 450 nm with a Bio-Rad 680 microplate reader (Bio-Rad Laboratories, Inc., Hercules, CA, USA).

### Cell invasion assay

Cells were cultured in normal medium overnight under hypoxic conditions. On the following day, 5×10^4^ cells were plated into the upper chamber wells of 24-well Biocoat Matrigel invasion chambers (8-*µ*m pores) (Becton-Dickinson, Franklin Lakes, NJ, USA), which were coated with Matrigel and maintained in 1% FBS F-12 medium under hypoxia or normoxia for 24 h. Non-invaded cells were removed. The invaded cells were fixed with 25% methanol and stained with crystal violet (Sigma-Aldrich). Cell numbers were counted in five random ×400 power fields under an inverted fluorescence light microscope [IX71; Olympus (Thailand) Co., Ltd., Bangkok, Thailand].

### Pathscan RTK signaling antibody array kit

The PathScan RTK Signaling Antibody Array kit (#7982; Cell Signaling Technology, Inc., Danvers, MA, USA) was used according to the manufacturer's instructions to simultaneously detect phosphorylation of 28 receptor tyrosine kinases and 11 important signaling molecules, including human epidermal growth factor receptor 2 (HER2) and -3, fibroblast growth factor receptor 2 (FGFR2), insulin receptor (InsR), Met, stem cell factor receptor (SCFR); ephrin type-A receptor 2 (EphA2), TEK tyrosine kinase, endothelial (TEK) and S6 ribosomal protein (S6RP). In brief, cells were washed with ice-cold 1X PBS and lysed in 1X Cell Lysis Buffer to collect cell lysates of normoxia vs. hypoxia (48 h) samples. The Array Blocking Buffer was added and lysates were incubated for 15 min at room temperature. The lysates were then added to the assay wells and incubated for 2 h at room temperature. Subsequently, the Detection Antibody Cocktail was added to each well and incubated for 1 h at room temperature followed by an addition of horseradish peroxidase-linked streptavidin and the slide was incubated for another 30 min at room temperature. The plates were then covered with LumiGLO/Peroxide reagent (Cell Signaling Technology, Inc.) and exposed to film (Kodak, Bangkok, Thailand) for 2–30 sec. The film was scanned using an Epson E11000XL-PH Photo Scanner (Epson, Suwa, Japan) and intensity was measured using ImageJ 1.47v (National Institutes of Health, Bethesda, MD, USA).

### Pathscan intracellular signaling array kit

The array kit (cat. no. 7323; Cell Signaling Technology, Inc.) was used according to the manufacturer's instruction. This kit allowed for the detection of phosphorylation or cleavage of 18 signaling molecules simultaneously, including ERK, heat shock protein 27 (HSP27), mammalian target or rapamycin (mTOR), B-cell lymphoma-2-associated death domain (Bad), c-Jun-N-terminal kinase (JNK), adenosine monophosphate-activated protein kinase (AMPK) and proline-rich Akt/PKB substrate 40 kDa (PRAS40). Cell lysates were collected after exposure to hypoxia for 48 h in HAM's F12 medium without fetal bovine serum and processed as described above.

### Inhibition of Met and ERK signaling

Inhibition of Met was performed using small hairpin (sh)RNA plasmids targeting Met (Santa Cruz Biotechnology, Inc., Dallas, TX, USA) according to the manufacturer's instructions. In brief, 2×10^5^ cells were plated in a six-well tissue culture plate in antibiotic-free normal growth medium until the cells were 70–80% confluent. Cells were then incubated with Lipofectamine (Invitrogen Life Technologies) together with the plasmids for 6 h at 37°C prior to replacement with fresh normal growth medium. Cells were then subjected to an invasion assay as described above. Block-iT Fluorescent Control (Invitrogen Life Technologies) was used to confirm transfection efficiency. For inhibition of ERK, cells were treated with U0126 (10 *µ*g/ml; Cell Signaling Technology, Inc.) overnight prior to the invasion assay.

### Human cholangiocarcinoma tissue samples

Cholangiocarcinoma tissue samples used in the present study were obtained from cholangiocarcinoma patients (n=44) who underwent surgical resection at Rajavithi Hospital (Bangkok, Thailand) from 2012–2014 (period of 18 months). The protocol of the present study was approved by the Ethics Committee of Rajavithi Hospital (Bangkok, Thailand).

### Immunohistochemical staining

Paraffin wax sections of cholangiocarcinoma specimens were dewaxed in xylene and transferred to various concentrations of alcohol (100, 85, 70 and 50%. Endogenous peroxidase activity was blocked with 0.5% hydrogen peroxide (Sigma-Aldrich) in methanol at room temperature for 10 min, then the sections were boiled in 10 mM citrate buffer (pH 6.0; Abcam) in a microwave oven for 10 min (750 W; LG MS-202W; Seoul, South Korea) for antigen retrieval. Non-specific binding was blocked by incubation with 3% normal horse serum (Gibco-BRL, Life Technology) for 20 min. Sections were incubated overnight at 4°C with the rabbit monoclonal antibody against HIF-1α (ab51608; 1:100 dilution; Abcam). Biotinylated goat anti-rabbit immunoglobulin G (E0432; 1:500 dilution; Dako, Glostrup, Denmark) was then added followed by an avidin-biotin-peroxidase conjugate (ABC Elite; Vector Laboratories, Burlingame, CA, USA) for 30 min at room temperature. The immunohistochemical reaction was developed with freshly prepared reagents from a Histofine SAB-PO kit (Nichirei Inc., Tokyo, Japan). The slides were then dehydrated with various concentrations of alcohol (95, 95, 100 then 100%) for 5 min each, then with xylene. The slides were then mounted. Sections were then visualized under high-power magnification (×400) using an Olympus BH2 microscope (field width, 0.5 mm; Olympus, Tokyo, Japan) and scored using the following three categories based on the percentage of positively stained cells: 1) negative, <5%; 2) weak, 5–50%; and 3) strong >50%.

### Statistical analysis

All experiments were performed in triplicate and values are expressed as the mean ± standard deviation. The student's *t*-test was used for analysis and P<0.05 was considered to indicate a statistically significant difference. The χ^2^ test was used for statistical analysis of the association between clinicopathological data and the expression of HIF-1α.

## Results

### Hypoxia induces HIF-1α protein expression

**Figure 1:**
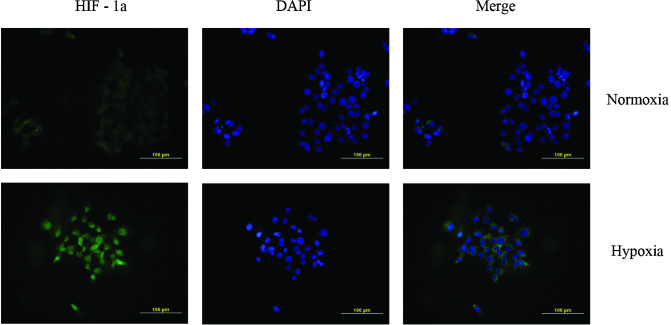
HIF-1α expression detected by immunofluorescence staining after hypoxia for 48 h (scale bar, 100 *µ*m). Cells cultured under hypoxia showed higher HIF-1α expression. HIF, hypoxia-inducible factor.

Immunofluorescence staining was performed to examine expression of HIF-1α under hypoxic conditions. An increased HIF-1α protein expression was observed after hypoxia for 48 h (Fig. 1). HIF-1α expression in the hypoxic group was detected in the nucleus as well as in the cytoplasm of RMCCA-1 cells.

### Hypoxia stimulates RMCCA-1 proliferation

**Figure 2:**
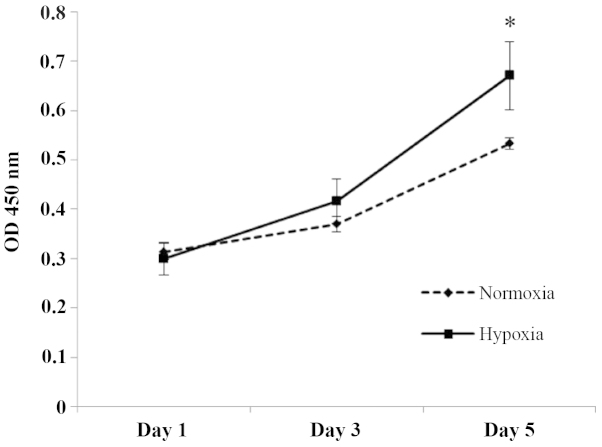
Effects of hypoxia on cell proliferation over the period of 5 days in RMCCA-1 cells are shown. (♦) Normoxia and (■) hypoxia; ^*^P<0.05 vs. normoxia. Values are expressed as the mean ± standard deviation (n=3). OD, optical density.

Cell proliferation of RMCCA-1 under hypoxic conditions was investigated over the course of 5 days. The results showed that hypoxia significantly stimulated RMCCA-1 cell growth (P<0.05) (Fig. 2). Cells cultured under hypoxia exhibited a higher growth rate and reached a statically significant difference compared with that of the normoxic cells on day 5.

### Hypoxia enhances RMCCA-1 invasion

**Figure 3:**
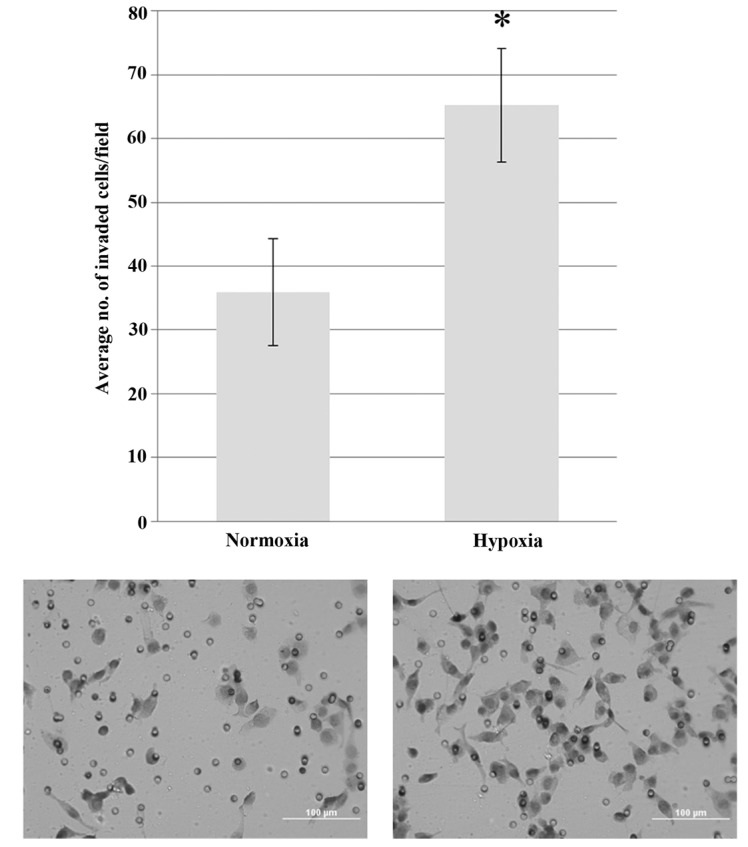
RMCCA-1 cell invasion *in vitro* after 24 h. The upper panel shows the average number of invaded cells per field, comparing between cells cultured under normoxic and hypoxic conditions. Values are expressed as the mean ± standard deviation (n=3). ^*^P<0.05 vs. normoxia. The lower panel shows representative images of the invasion assay (scale bar, 100 *µ*m).

Several studies have reported that hypoxia promotes cancer cell invasion (5,7). The present study performed a Transwell invasion assay to examine the effect of hypoxia on the invasion of RMCCA-1 cells. After 24 h, the number of invaded cells was significantly increased under hypoxic conditions compared with that following incubation under normoxic conditions (P<0.05) (Fig. 3).

### Induction of Met under hypoxia

**Figure 4:**
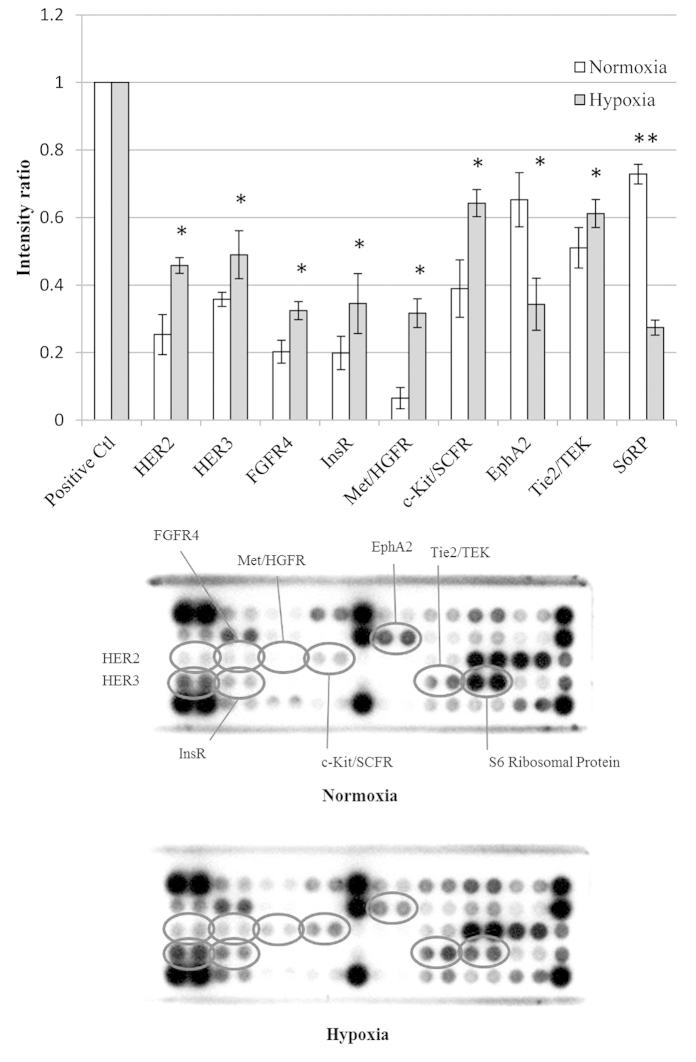
Effects of hypoxia on phosphorylation of signaling proteins. Lower panel shows a representative pair of chemiluminescent images produced using the PathScan RTK Signaling Antibody Array kit. The upper panel shows the pixel intensity ratio of phosphorylated signaling molecules. ^*^P<0.05, ^**^P<0.001 for the comparison between hypoxia and normoxia; values are presented as the mean ± standard deviation. Ctl, control; HER2, human epidermal growth factor receptor 2; FGFR2, fibroblast growth factor receptor 2; InsR, insulin receptor; Met/HGFR, hepatocyte growth factor receptor; SCFR, stem cell factor receptor; EphA2, ephrin type-A receptor 2; TEK, TEK tyrosine kinase, endothelial; S6RP, S6 ribosomal protein.

In order to elucidate signaling pathways and receptors involved in hypoxia, the phosphorylation of various RTKs and signaling molecules was examined using the Pathscan RTK signaling antibody array kit. It was found that phosphorylation of a number of signaling molecules, including HER2, HER3, FGFR4, InsR, Met/HGFR, c-Kit/SCFR and Tie2/TEK in hypoxic cells was markedly increased compared with that in normoxic cells (P<0.05) (Fig. 4). In addition, a significant decrease in the phosphorylation levels of EphA2 and S6RP was observed (P<0.05).

### Activation of ERK in hypoxia

**Figure 5:**
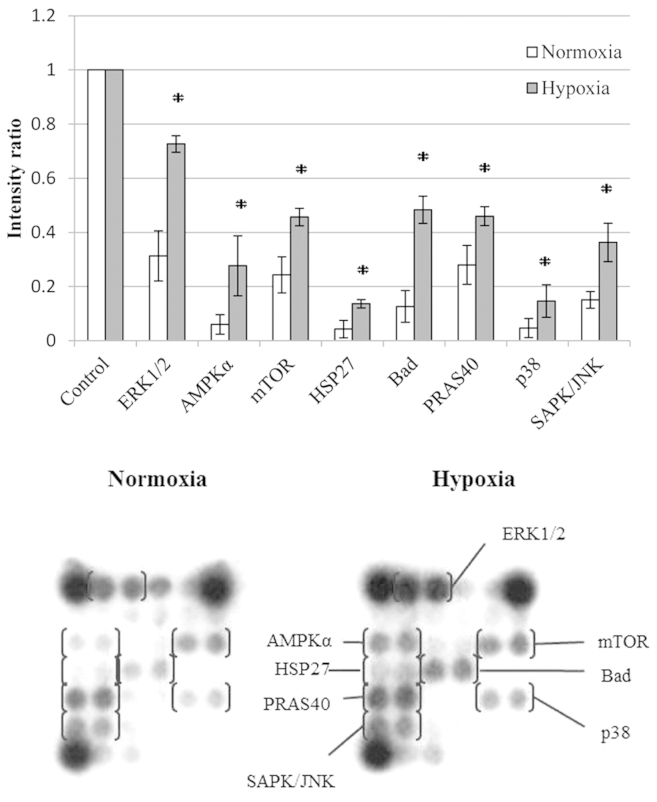
Activation of intracellular signaling nodes by hypoxia. The lower panel presents chemiluminescent images produced with the PathScan Intracellular Signaling Array kit. The upper panel shows the pixel intensity ratio of activated signaling nodes. ^*^P<0.05, normoxia vs. hypoxia; values are presented as the mean ± standard deviation. ERK, extracellular signal-regulated kinase; HSP, heat shock protein; mTOR, mammalian target or rapamycin; Bad, B-cell lymphoma-2-associated death domain; JNK, c-Jun-N-terminal kinase; AMPK, adenosine monophosphate-activated protein kinase; PRAS40, proline-rich Akt/PKB substrate 40 kDa.

The present study further investigated downstream effectors of Met, which are associated with hypoxia-induced invasion. The Pathscan intracellular signaling array kit was used to determine changes in phosphorylation or cleavage of several intracellular signaling molecules. The results showed an increase in the phosphorylation levels of ERK1/2, AMPKα, mTOR, HSP27, Bad, PRAS40, p38 and SAPK/JNK in the hypoxic cells compared with those in normoxic cells (P<0.05) (Fig. 5).

### Inhibition of Met and ERK attenuates hypoxia-induced invasion

**Figure 6:**
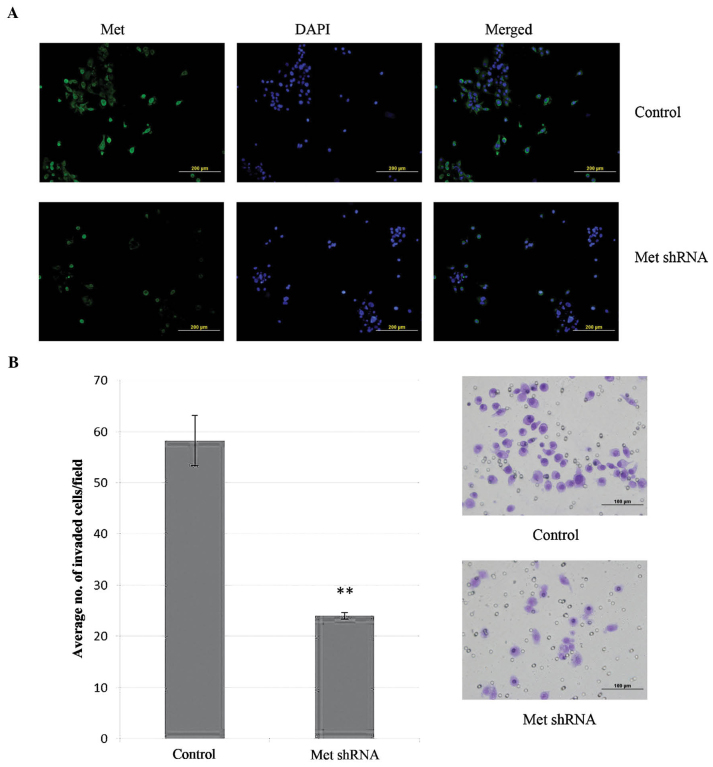
Effect of inhibition of Met expression on RMCCA-1 invasion under hypoxia. (A) Immunofluorescence staining of Met protein in RMCCA-1 cells (scale bar, 100 *µ*m). (B) Cell invasion capabilities after treatment with Met shRNA vs. control under hypoxic conditions (scale bar, 100 *µ*m). Values are expressed as the mean ± standard deviation. ^**^P<0.001 vs. control. shRNA, small hairpin RNA; Met, hepatocyte growth factor receptor.

**Figure 7:**
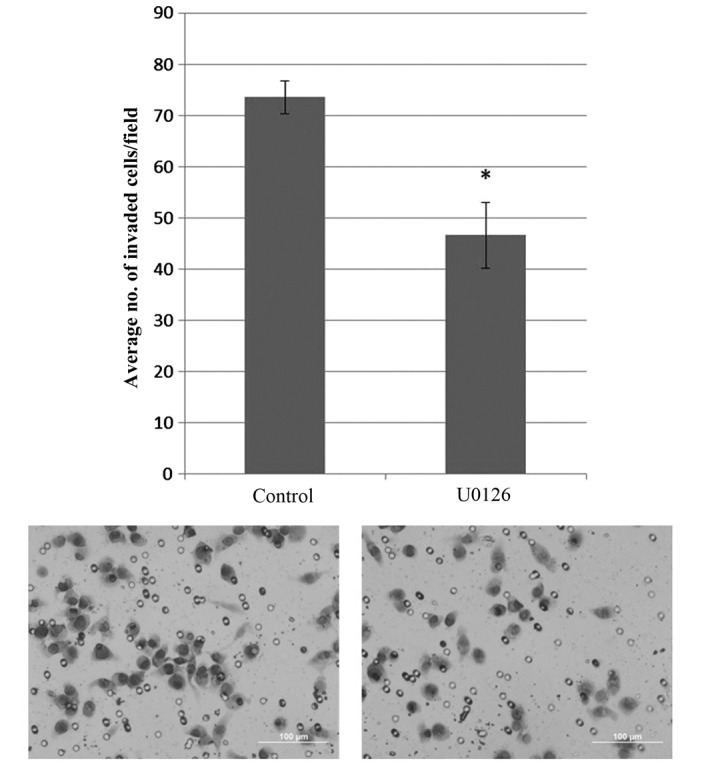
Effects of extracellular signal-regulated kinase inhibition on cell invasion under hypoxic conditions. Cell invasion capabilities after treatment with U0126 (10 *µ*g/ml) for 24 h (scale bar, 100 *µ*m). Values are expressed as the mean ± standard deviation. ^*^P<0.05 vs. control.

Given that Met exhibited the greatest change in its phosphorylation levels among the proteins analyzed, the involvement of Met in hypoxia-induced invasion was assessed by silencing Met expression in RMCCA-1 cells. Immunofluorescence staining was used to confirm a decrease in Met expression after transfection (Fig. 6A). The results showed that the invasion ability of RMCCA-1 was significantly reduced in cells treated with shRNA compared to cells treated with control small interfering RNA (P<0.001) (Fig. 6B). Since ERK is one of the main downstream effectors of Met and it has been demonstrated to have an important role in metastasis (15,20), it was hypothesized that Met-induced invasion of RMCCA-1 cells under hypoxia was mediated through ERK. To investigate whether ERK activation was responsible for increased invasion, U0126 was used to inhibit ERK. A significant decrease in cell invasion was identified in cells treated with U0126 compared to that of the control (P<0.05) (Fig. 7). The results therefore suggested that activation of the Met/ERK pathway is critical for the hypoxia-induced invasion of RMCCA-1 cells.

### *HIF-1α expression in paraffin-embedded* cholangiocarcinoma *samples*

**Figure 8:**
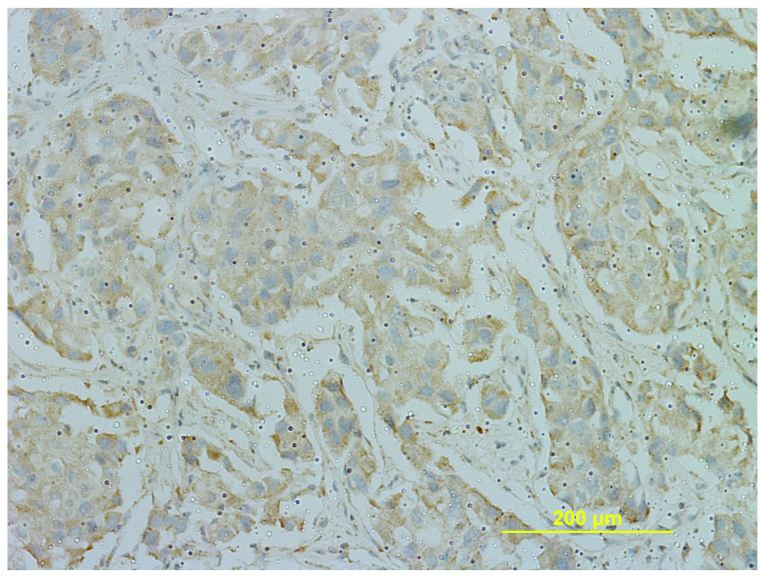
Representative image of immunohistochemical analysis of HIF-1α expression in cholangiocarcinoma samples. Positive staining for HIF-1α (brown) in choloangiocarcinoma cells (scale bar, 200 *µ*m). HIF, hypoxia-inducible factor.

The expression of HIF-1α was determined by immunohistochemistry in 44 paraffin-embedded cholangio-carcinoma specimens (Fig. 8). In these cancerous tissues, HIF-1α-specific signals were localized mainly in the cytoplasm of cholangiocarcinoma cells but not in normal biliary epithelial cells. It was found that 66.0% (n=29/44) of the cholangiocarcinoma specimens were strongly positive for HIF-1α expression. The expression of HIF in cholangiocarcinoma was detected in all stages of the disease. However, the expression levels were not significantly correlated with lymph node or metastatic status (Table I).

## Discussion

The chronic hypoxia within solid tumors provides a selective pressure for cells with a more aggressive phenotype to survive. Thus, a better understanding of cellular adaptation to low oxygen tension is crucial for the development of improved treatment strategies. The contribution of hypoxia to tumor metastasis has been reported in several types of cancer, including gliomas (11), breast cancer (10) and hepatocellular carcinoma (5). In the present study, an *in vitro* experiment demonstrated that hypoxia enhanced the proliferation and invasion of cholangiocarcinoma. In addition, the present study suggested that hypoxia-induced invasion is associated with increased phosphorylation of Met and activation of the MAPK/ERK signaling pathway. The findings are in agreement with previous studies suggesting that hypoxia promotes metastatic progression (5–11). The results of the present study suggested that the hypoxic environment promotes the malignant characteristics of cholangiocarcinoma.

The present study first examined the induction of HIF-1α under hypoxic conditions using immunofluorescence staining and found higher HIF-1α expression in hypoxic cells, showing HIF-1α stabilization under hypoxia. The presence of HIF-1α was observed in the nucleus as well as in the cytoplasm of RMCCA-1, indicating nuclear translocation for regulating gene transcription.

A cell proliferation assay was then used to identify the effects of hypoxia on tumor growth. Studies on the association between hypoxia and cell proliferation have been contradictory in recent years. Hypoxia was found to inhibit cell proliferation in certain cell types (5), while promoting cell growth in others, suggesting a cell-specific variation in response to hypoxic stress. This is possibly due to genetic variation among cancer cell populations. The results of the present study implied that RMCCA-1 cultured under hypoxic conditions proliferated more efficiently compared to those cultured under normoxic conditions. These findings were discordant with a study by Seubwai *et al* (21), which reported suppressed growth after exposure of various cholangiocarcinoma cell lines to hypoxia. The present study reported increased cell proliferation induced by hypoxia, supporting that hypoxia regulated the expression of genes involved in glycolysis and accelerated cell growth. Gwak *et al* (22) reported that hypoxia enhanced hepatocellular carcinoma cell growth through induction of hexokinase II (22). The levels of HIF-1α were correlated with phosphoinositide-dependent kinase-1, lactate dehydrogenase A and pyruvate kinase, muscle 2 expression and inhibition of HIF-1α repressed pancreatic cancer cell growth (23). However, further studies are required to identify proteins responsible for accelerated cell growth following exposure of RMCCA-1 to hypoxia.

Furthermore, the present study found that the invasion was significantly increased as a result of hypoxic stimulation. Alterations in phosphorylation/activation of important signaling molecules were subsequently examined in an attempt to identify pathways responsible for hypoxia-induced invasion. Hypoxia is known to mediate various signaling cascades. The antibody array kit revealed significantly increased phosphorylation of HER2, HER3, FGFR4, InsR, Met/HGFR, c-Kit/SCFR and Tie2/TEK. The greatest change in phosphorylation was observed in Met/HGFR. The results of the intracellular signaling array kit assay further revealed increased phosphorylation of a number of intracellular signaling nodes, including ERK1/2, AMPKα, mTOR, HSP27, Bad, PRAS40, p38 and SAPK/JNK.

A previous study demonstrated an involvement of Met in cholangiocarcinoma cell lines by showing that inhibition of Met and ERK activation significantly reduced cell invasion abilities (15). It was therefore hypothesized that activation of the ERK pathway by Met may be the molecular event contributing to the malignant phenotype of RMCCA-1 under hypoxic conditions. To confirm this hypothesis, Met expression was inhibited using shRNA and ERK expression was blocked by treatment with U0126, a MAPK kinase (MEK) inhibitor. The results showed that the increase in cell invasion under hypoxia was abrogated following blocking of Met or ERK.

Met is a well-known receptor that has a significant role in tumor progression in various cancers, including cholangiocarcinoma (24). Overexpression of this receptor was reported in numerous types of solid tumor and is associated with malignant disease (25). Stimulation of Met leads to the activation of multiple downstream signaling pathways with two major pathways being the MAPK/ERK and the PI3K/Akt signaling axes (20). In the present study, the antibody signaling array kits revealed increased phosphorylation of ERK1/2 but not Akt in the presence of hypoxic stress. ERK is a downstream signaling molecule activated by MEK in response to growth stimuli and involved in multiple signaling pathways regulating cell survival, proliferation and motility (7). The MAPK pathway has been shown to have a role in HIF-1α phosphorylation to enhance its transcriptional activity (26). In addition, there is evidence suggesting that activation of ERK is associated with hypoxia-induced metastasis (7,27).

A link between hypoxia and Met overexpression in hypoxia-induced invasion has been demonstrated in a number of studies (28–31). It was shown that Met expression can be induced during hypoxia at the transcriptional level in a HIF-1α-dependent manner (29). However, the molecular mechanisms of Met activation by hypoxia in the present study as well as the precise association between tumor hypoxia and cell invasion in cholangiocarcinoma still remain to be fully elucidated. In the present study, induction of HIF-1α was observed when RMCCA-1 cells were exposed to chronic hypoxia. This may be linked with increased activation of the MAPK cascade, which in turn results in upregulation of various genes by HIF-1.

In addition, immunohistochemical analysis indicated that HIF-1α is preferentially expressed in cholangiocarcinoma but not in the adjacent normal bile duct tissues obtained through biopsy from cholangiocarcinoma patients. However, the expression levels of HIF-1α were not significantly correlated with either tumor differentiation or staging status. This finding is in contrast to a study on gastric cancer, which found that the expression of HIF-1α was significantly correlated with the clinical staging (32). Variations in the biological features of the tumors and the limited number of specimens in the present study may account for different results.

In conclusion, the present study reported that hypoxic stress enhanced the aggressive phenotype of cholangiocarcinoma. It was shown that the molecular basis underlying hypoxia-induced invasion is through the activation of the Met and the ERK signaling pathway. These findings supported Met as one of the potential targets in therapeutic approaches to treat cholangiocarcinoma.

## Figures and Tables

**Table I:** Association between HIF-1α expression and clinicopathological features in cholangiocarcinoma samples.

		HIF-1α expression		
Variable	Total (n=44)	Weak	Strong	P-value^a^
Age (years)				
<60	23	8	15	0.919
>60	21	7	14	
Sex				
Male	19	7	12	0.737
Female	25	8	17	
TNM stage^b^				0.198
I/II	17	8	9	
III/IV	27	7	20	
Tumor differentiation				0.107
Well	40	12	28	
Moderate/poor	4	3	1	

a χ^2^ test.

b Union for International Cancer Control. TNM, tumor, nodes and metastasis score. HIF, hypoxia-inducible factor.
